# Exploring the anti-fibrotic effects of safflower in systemic sclerosis based on metabolomics and gut microbiota analyses

**DOI:** 10.3389/fphar.2026.1719219

**Published:** 2026-02-18

**Authors:** Yu Zhang, Xingfeng Ping, Chao Wang, Xueyan Zhou, Yi Huang, Kai Li, Junying Lv

**Affiliations:** 1 Department of First Clinical Medical College, Guangxi Medical University, Nanning, Guangxi, China; 2 Department of Traditional Chinese Medicine, The First Affiliated Hospital of Guangxi Medical University, Nanning, Guangxi, China

**Keywords:** fibrosis, gut microbiota, metabolomics, safflower (Carthamus tinctorius L.), scleroderma (or systemic sclerosis)

## Abstract

**Background:**

Systemic sclerosis (SSc) is a complex autoimmune disease characterized by inflammation, fibrosis, and multi-organ involvement. Traditional botanical drugs have long been used empirically to manage fibrotic and inflammatory conditions, yet their pharmacological effects remain incompletely understood.

**Objective:**

The aim of this study was to investigate the effects of an aqueous extract of safflower on fibrotic and inflammatory phenotypes, gut microbiota composition, and fecal metabolic profiles in a bleomycin-induced murine model of systemic sclerosis.

**Methods:**

A murine model of systemic sclerosis (SSc) was established by bleomycin (BLM) induction, followed by safflower intervention for 28 days. The therapeutic effects of safflower on SSc-associated fibrosis and inflammation were evaluated by hematoxylin–eosin (HE) staining, Masson’s trichrome staining, collagen fiber quantification, and enzyme-linked immunosorbent assay of inflammatory mediators (IL-1β, IL-13, and Lipopolysaccharide). Gut microbiota composition and fecal metabolic profiles were analyzed using 16S rRNA gene sequencing and untargeted UHPLC–QTOF–MS-based metabolomics to explore the potential mechanisms associated with the anti-fibrotic effects of safflower in systemic sclerosis.

**Results:**

Safflower aqueous extract alleviated bleomycin-induced systemic sclerosis phenotypes, including dermal sclerosis, multi-organ collagen deposition, and inflammatory responses in skin, lung, and colon tissues. Untargeted fecal metabolomics revealed that safflower intervention significantly modulated metabolic pathways primarily related to central carbon metabolism, amino acid and protein metabolism, and steroid hormone biosynthesis. Furthermore, 16S rRNA gene sequencing demonstrated that safflower partially restored gut microbial richness and diversity and reversed systemic sclerosis–associated dysbiosis by increasing the relative abundance of putatively beneficial taxa (e.g., *Cyanobacteria*, *Chloroflexi*, *Acidobacteria*, and *Nitrospirae)* while reducing inflammation-associated genera, including *Deferribacteres*, *Odoribacter*, *Akkermansia*, *Helicobacter*, and Prevotellaceae*_NK3B31_group*. These results suggest that the anti-fibrotic effects of safflower are associated with integrated modulation of gut microbiota and metabolic profiles.

**Conclusion:**

Safflower alleviated fibrotic and inflammatory phenotypes in systemic sclerosis, which may be associated with the modulation of gut microbiota composition and metabolic homeostasis.

## Introduction

Systemic sclerosis (SSc), also known as scleroderma, is a complex autoimmune connective tissue disease characterized by immune dysregulation, vasculopathy, and progressive fibrosis affecting multiple organs, including the skin, lungs, gastrointestinal tract, heart, and skeletal muscle (Allanore et al., 2015; Orlandi et al., 2020; Orlandi et al., 2022). Recent global epidemiological studies estimate the prevalence of systemic sclerosis to be approximately 17.6–18.9 per 100,000 individuals, with incidence rates of about 1.4 per 100,000 person-years (Bairkdar et al., 2021; Tian et al., 2023). Clinically, SSc is classified into limited cutaneous systemic sclerosis (lcSSc) and diffuse cutaneous systemic sclerosis (dcSSc) based on the extent of skin involvement, with the diffuse subtype generally associated with more rapid disease progression and higher mortality (LeRoy et al., 1988; Cossu et al., 2017; Orlandi et al., 2022). Although the precise etiology of SSc remains incompletely understood, accumulating evidence indicates that aberrant immune activation, endothelial dysfunction leading to vasculopathy, and excessive fibroblast activation with extracellular matrix deposition jointly drive disease pathogenesis (Orlandi et al., 2020; Denton and Khanna, 2017; Orlandi et al., 2022). Current Western medical treatments primarily rely on glucocorticoids and immunosuppressants; however, their clinical utility is limited by restricted efficacy and substantial adverse effects.

Safflower (*Carthamus tinctorius L.*), a representative traditional Chinese medicine (TCM) used for promoting blood circulation and removing blood stasis, was first recorded in the Kaibao Bencao over 1,000 years ago. It contains multiple bioactive metabolites and exhibits pharmacological properties such as vasodilation, immunomodulation, and anti-fibrotic effects (Wu et al., 2021). Cui et al. reported that safflower polysaccharides exert vascular protective effects in steroid-induced femoral head necrosis (Cui et al., 2020). Fu et al. reported that hydroxysafflor yellow A (HSYA) attenuates hepatic fibrosis in a mouse model of liver cancer by degrading collagen and modulating the gut microbiota to enhance immune infiltration (Fu et al., 2021). Our previous research confirmed that safflower aqueous extract downregulates inflammatory factors, collagen, and α-smooth muscle actin in SSc model mice, suppresses immune inflammation, reduces myofibroblast activation and extracellular matrix production, and alleviates skin and pulmonary fibrosis (Hu and Lü, 2018; Li et al., 2022).

In recent years, increasing attention has been paid to the role of the gut microbiota in maintaining intestinal homeostasis, regulating host immune responses, and modulating inflammatory processes. Accumulating evidence links gut microbiota dysbiosis to autoimmune disorders, including rheumatoid arthritis, systemic lupus erythematosus, and ankylosing spondylitis (Scher et al., 2013; Hevia et al., 2014; Klingberg et al., 2019). Emerging studies further suggest that gastrointestinal involvement in SSc may be associated with alterations in gut microbial composition, which could contribute to immune imbalance and fibrotic progression (Teaw et al., 2021; Bellando-Randone et al., 2024). Nevertheless, mechanistic evidence linking gut microbiota alterations to SSc pathogenesis remains limited and warrants further investigation. In China, the use and therapeutic efficacy of safflower in treating SSc are well recognized (Yang and Lyu, 2013). Recent pharmacological reviews have summarized its blood activating, anti-inflammatory, and immuimmunoregulatory ne regulating effects, which have been widely applied in various diseases and support its therapeutic potential (Bai et al., 2025).

Fecal metabolomics, as an important branch of metabolomics, enables comprehensive evaluation of host–microbiota interactions and metabolic states through the analysis of fecal metabolites. Recent studies have highlighted the value of integrating metabolomic and microbiome data to elucidate disease mechanisms and to assess therapeutic interventions, particularly in immune-mediated and fibrotic diseases (Schrimpe-Rutledge et al., 2016; Morales-González et al., 2023; Bhosle et al., 2022). In this context, we employed 16S rRNA gene sequencing to investigate gut microbiota alterations in SSc and to assess the effects of safflower on microbiota composition, inflammatory cytokines, and endotoxin levels. Furthermore, fecal metabolomic analysis was performed in bleomycin-induced SSc mice with and without safflower treatment to explore potential safflower-associated microbiota-metabolite interaction mechanisms and to provide new insights into complementary therapeutic strategies for systemic sclerosis.

## Materials and methods

### Drugs, reagents, and instruments

Safflower (*C. tinctorius L.*) was sourced from Guangdong Kangmei Pharmaceutical Co., Ltd. (Batch No. 190701921) and provided by the TCM Pharmacy of the First Affiliated Hospital of Guangxi Medical University. The botanical drug was authenticated as Carthamus tinctorius L. by Prof. Zhengguang Huang, a licensed pharmacist at the Department of Pharmacy, the First Affiliated Hospital of Guangxi Medical University, based on macroscopic and microscopic characteristics according to the Chinese Pharmacopoeia. A voucher specimen was deposited at the TCM Pharmacy of the First Affiliated Hospital of Guangxi Medical University. Compliance with the ConPhyMP guidelines, including detailed botanical authentication, extraction procedures, and reporting checklist items, is provided in Supplementary Data Sheets 2, 3. Bleomycin hydrochloride for injection was purchased from Zhejiang Hisun Pharmaceutical Co., Ltd. (Batch No.: H20055883). Fecal genomic DNA extraction and purification kits (Beijing Tiangen Biotech Co., Ltd.); ELISA kits for IL-1β, IL-13, and LPS (Jiangsu Bosheng Biotechnology Co., Ltd., Batch Nos.: BS-E8668M1, BS-E8661M1, BS-E9334M1); High-Fidelity PCR Premix (NEB, United States, Batch No.: M0532S). Equipment included: Bio-Rad T100 Gradient PCR Cycler (Bio-Rad, United States), DYY-6C electrophoresis unit (LiuYi Instrument Factory, Beijing), Invitrogen Qubit 3.0 Fluorometer (United States), Agilent 2100 Bioanalyzer (United States), NovaSeq 6000 sequencing system (Illumina, United States), hematoxylin-eosin (HE) and Masson’s trichrome staining kits (Wuhan Servicebio, Batch Nos.: G1003, G1006), and OLYMPUS BX53 upright fluorescence microscope (Olympus, Japan). AB SCIEX TripleTOF 6600 mass spectrometer, Agilent 1290 Infinity UHPLC system, refrigerated centrifuge (Eppendorf 5430R). Detailed information regarding botanical authentication, source, batch information, and compliance with pharmacopoeial and ConPhyMP reporting requirements is provided in Supplementary Data Sheet 2.

### Preparation of safflower aqueous extract

A traditional aqueous extract of safflower *(C. tinctorius L.)* was prepared under standardized conditions with reference to previously reported methods (Chen, 2007; Guo and Jia, 1996). First, 15.0 g of dried safflower were accurately weighed and extracted with 1000 mL of ultrapure water. After soaking for 1 h, the mixture was heated to boiling and extracted for 1 h. The first extract was filtered and collected. The residue was then re-extracted with an additional 1000 mL of ultrapure water under identical conditions for 1 h. The second extract was filtered and combined with the first. The combined aqueous extracts were concentrated to a viscous extract under reduced pressure at 65 °C using a rotary evaporator. The concentrate was subsequently freeze-dried, yielding 3.71 g of a lyophilized safflower aqueous extract powder, with an extraction yield of approximately 24.7% (w/w), representing the mean yield obtained for this batch under identical extraction conditions. For animal administration,a homogeneous dosing suspension was freshly prepared each day. Briefly, 111.3 mg of the lyophilized extract powder was accurately weighed and initially dispersed in 1 mL of ultrapure water by vigorous vortex mixing. The resulting suspension was then quantitatively transferred to a volumetric flask and brought to a final volume of 3 mL with the same solvent, followed by thorough vortex mixing to obtain a homogeneous suspension at a final concentration of 37.1 mg/mL for subsequent use. The chemical profile of the aqueous extract was characterized by UPLC-QTOF-MS, and representative total ion chromatograms (TICs) acquired in both positive and negative ionization modes, together with qualitative identification of major constituents, are provided in the Supplementary Data Sheets 1, 3.

### Animals and drug administration

A cohort of 48 specific pathogen-free (SPF) grade female BALB/c mice, each weighing between 18 and 22 g, was procured from Hunan SJA Laboratory Animal Co., Ltd. [License No. SCXK (Xiang) 2016-0002]. The mice were maintained in an SPF laboratory environment under standardized conditions, including a temperature of 25 °C ± 2 °C and a relative humidity of 50% ± 5%, with unrestricted access to food and water. After a 7-day acclimatization period, a total of 48 mice were randomly allocated into four groups (n = 12 per group) using simple randomization based on a computer-generated random number sequence. The groups included the Control, SSc Model, Prednisolone acetate (Pred), and Safflower (Saff). Prednisolone was included as a reference treatment because of its well-established anti-inflammatory and anti-fibrotic effects and its clinical relevance in systemic sclerosis. This provides a clinically relevant benchmark for evaluating the therapeutic efficacy of safflower. All mice except the Control group were administered subcutaneous injections of bleomycin phosphate buffer (200 μg/mL, 0.1 mL/day) in the dorsal region for 28 days (Morin et al., 2015); the Control group received 0.1 mL/d of 0.01 mol/L PBS instead. Successful model induction was defined by signs of dermal sclerosis, dermal thickening, uniform collagen bundle accumulation, and lung fibrosis. Histopathological inflammation scoring of the skin, lung, and colon was defined *a priori* as an objective criterion for successful model establishment. The inflammation score was significantly higher than that of the control group, accompanied by an increase in skin thickness and collagen deposition, the model was successfully confirmed; Animals that do not meet these standards are excluded. Drug dosages were calculated based on mice with an average body weight of 20 g and a fixed daily gavage volume of 0.2 mL (equivalent to 10 mL/kg). Accordingly, mice in the Pred group received prednisolone acetate by oral gavage at a dose of 4.5 mg/kg/day. Mice in the Saff group received the safflower aqueous extract by oral gavage at a fixed volume of 0.2 mL per day. The dosing suspension had a working concentration of 37.1 mg/mL of freeze-dried safflower aqueous extract, which corresponds to 0.15 g crude botanical drug equivalent per mL based on the experimentally determined extraction yield (24.7%, w/w). Under these conditions, the accurate daily dose administered to the Saff group was 371 mg of freeze-dried extract per kg body weight per day. The crude botanical drug equivalent value (1.5 g/kg/day) is provided solely as a reference unit, whereas all pharmacological dosing calculations and interpretations in the present study are based on the actual amount of freeze-dried extract administered. Mice in both the Control and Model groups received an equal volume of physiological saline once daily. All treatments were administered once daily for 28 consecutive days.

### Sample collection and processing

To ensure a stable metabolic state and reduce dietary effects, a standardized procedure was followed before collecting fecal samples. Twelve hours after the last dose, mice were moved to clean cages and fasted with water available. Fresh feces were collected 12–24 h post-fasting, snap-frozen in liquid nitrogen, and stored at −80 °C for later analysis. After administration of 2% sodium pentobarbital (50 mg/kg, intraperitoneal) for anesthesia, retro-orbital bleeding was performed to collect blood samples. After a 2-h incubation at room temperature, the blood samples were subjected to centrifugation at 2000 rpm for 15 min at 4 °C to isolate serum for ELISA analysis. At the injection site, dorsal skin, together with lung tissue and a 1–2 cm segment of colon (rinsed in pre-cooled PBS), was harvested and preserved in 4% paraformaldehyde for 48 h. Samples were then dehydrated, embedded in paraffin, and sectioned (4 μm) for histological analysis. Remaining colonic tissue was snap-frozen in liquid nitrogen for ELISA analysis.

### Observation indicators and methods

#### Clinical observation

During the modeling period, the general condition including activity, appetite, appearance, responsiveness, skin elasticity, and fecal characteristics was recorded daily in all groups.

### Histopathology

To reduce observational bias, tissue sections were blinded by an independent researcher who de-identified and randomly coded them. As previously described (Liu J. J. et al., 2019), two pathologists, unaware of the experimental groups, independently evaluated hematoxylin–eosin (HE) and Masson’s trichrome–stained sections of skin, lung, and colon to assess pathological changes and collagen fiber deposition. The final analysis used the average scores from both observers. Histopathological inflammation scores were used as *a priori* criteria to evaluate successful model establishment (Park et al., 2022). In parallel, collagen deposition was quantitatively assessed by collagen volume fraction (CVF) based on Masson’s trichrome staining, serving as a complementary indicator of fibrotic severity (Chen et al., 2025).

#### ELISA assays

ELISAs were conducted following the manufacturer’s guidelines. Samples (serum and colon tissue homogenates) were re-coded with random identifiers to ensure the technician was blinded to group assignments. Under these conditions, IL-1β, IL-13, and LPS concentrations were measured, with cytokine levels in colon tissue homogenates normalized to total protein content and expressed as pg/mg protein.

#### 16S rRNA high-throughput sequencing

Under strict blinding protocols, where samples were randomly coded and group assignments were hidden until final analysis, microbiome analysis was conducted using a standardized pipeline for reproducibility and quality control. Genomic DNA was extracted from 200 mg of fecal samples, verified for purity, and normalized to 1 ng/μL. The V3–V4 region of the 16S rRNA gene was amplified using primers 341F (5′-CCTAYGGGRBGCASCAG-3′) and 806R (5′-GGACTACNNGGGTATCTAAT-3′). Amplicons were purified, and sequencing libraries were prepared with the TruSeq® DNA PCR-Free Kit. Library quality was checked with a Qubit 3.0 Fluorometer and an Agilent 2100 Bioanalyzer before paired-end sequencing (2 × 250 bp) on an Illumina NovaSeq 6000. Following demultiplexing, raw reads underwent stringent quality control, which included read filtering via the QIIME pipeline (truncating reads at the first base with a quality score ≤19 in a window of ≥3 consecutive low-quality bases, removing reads containing ambiguous bases or failing length thresholds), chimera removal using the UCHIME algorithm against the Gold database, and sequence depth normalization across all samples based on the sample with the lowest read count to ensure comparability. High-quality sequences were clustered into operational taxonomic units (OTUs) at a 97% similarity threshold using UPARSE, with representative sequences taxonomically annotated against the SILVA SSU rRNA database (release 138). All subsequent downstream analyses—including microbial composition profiling, alpha- and beta-diversity calculations, principal coordinate analysis (PCoA), and differential taxa analysis—were performed on the Majorbio Cloud Platform. The entire experimental and bioinformatic workflow was conducted by Guangxi Profile Information Technology Co., Ltd.

### Metabolomics analysis

Fecal metabolomic analysis was performed using a high-resolution untargeted approach. Frozen samples were thawed, mixed with pre-cooled methanol/acetonitrile/water, vortexed, ultrasonication, incubated at −20 °C, and centrifuged. The supernatant was vacuum-dried, reconstituted in acetonitrile/water, vortexed, centrifuged again, and the resulting supernatant was injected for mass spectrometric analysis.

Chromatographic separation was performed on an Agilent 1290 Infinity LC system with a HILIC column (Waters ACQUITY UPLC BEH Amide, 1.7 µm, 2.1 × 100 mm) at 25 °C, with a flow rate of 0.5 mL/min and an injection volume of 2 µL. The mobile phase included water with 25 mM ammonium acetate and 25 mM ammonium hydroxide (solvent A) and acetonitrile (solvent B), following a gradient elution (see Table 1).

**TABLE 1:** Gradient elution program.

Time (min)	Mobile phase B	Mobile phase A
0–0.5	95%	5%
0.5–7.0	95–65%	5%–35%
7.0–8.0	65%–40%	35%–60%
8.0–9.0	40%	60%
9.0–9.1	40%–95%	60%–5%
9.1–12.0	95%	5%

Mass spectrometric detection was conducted using an AB SCIEX TripleTOF 6600 mass spectrometer operated in both positive and negative electrospray ionization (ESI) modes. Ion source settings included: nebulizing gas at 60, curtain gas at 30 psi, temperature at 600 °C, and spray voltage at ±5500 V. The full-scan mass range was m/z 60–1000 with a 0.20 s per spectrum accumulation time. For MS/MS in data-dependent acquisition mode, the scan range was m/z 25–1000 with a 0.05 s/spectrum accumulation time, and collision energy at 35 ± 15 eV.

To ensure data quality and control batch effects, rigorous quality control (QC) procedures were implemented. A pooled QC sample, generated by combining equal aliquots of all biological samples, was injected at regular intervals throughout the analytical sequence to monitor instrument stability and analytical reproducibility. Samples were analyzed in a randomized order to reduce bias. Raw data were converted to mzXML format using Proteo Wizard and processed with XCMS for peak detection and alignment. Data quality was assessed based on QC sample stability, with necessary corrections applied. Metabolites were identified by matching against an in-house database using retention time, accurate mass, and MS/MS fragmentation patterns, with manual verification to ensure Metabolomics Standards Initiative (MSI) Level 2 confidence or higher.

### Statistical analysis

Statistical analysis were performed using SPSS version 22.0. Data were tested for normality using the Shapiro–Wilk test and for homogeneity of variance using Levene’s test prior to parametric analysis. Data with normal distribution were presented as mean ± standard deviation. One-way analysis of variance (ANOVA) was used for multiple group comparisons, followed by LSD *post hoc* tests. Spearman’s rank correlation was performed to assess relationships between gut microbiota and metabolites, with significance set at P < 0.05.

## Results

### Effects of safflower on clinical symptoms in SSc mice

**FIGURE 1:**
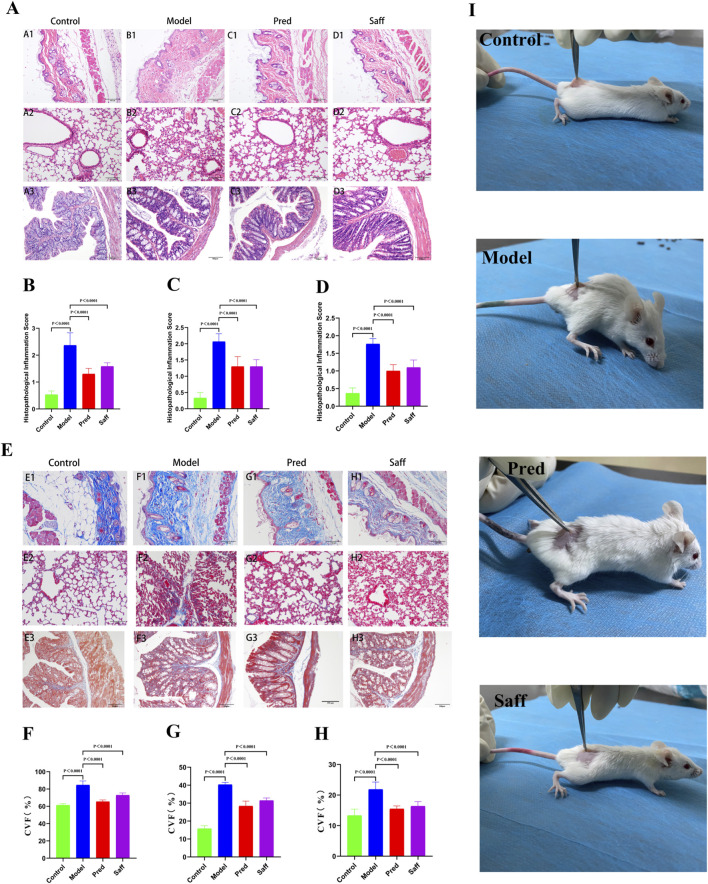
Clinical manifestations, inflammatory responses, and fibrosis in model mice. **(A)** HE staining (Skin: A1–D1; Lung: A2–D2; Colon: A3–D3). **(B–D)** Semi-quantitative histopathological inflammation scores (Skin: B; Lung: C; Colon: D). **(E)** Masson staining (Skin: E1–H1; Lung: E2–H2; Colon: E3–H3). **(F–H)** Quantitative analysis of collagen volume fraction (CVF) (Skin: F; Lung: G; Colon: H). **(I)** Gross clinical features of mice from each group, including skin appearance, posture, and overall condition. Data are presented as mean ± SD. Statistical analyses were performed using one-way ANOVA followed by LSD *post hoc* tests. Statistical significance is indicated by exact P-values or conventional significance thresholds. For comparisons yielding extremely small P-values, results are denoted as P < 0.0001. P < 0.0001 compared with the Model group.

As shown in Figure 1I, mice in the Control group displayed a good general condition, high responsiveness, agility, shiny and smooth fur, and intact, soft, and elastic dorsal skin. In contrast, mice in the Model group showed poor mental status, sluggish response, sluggish movement, dull fur, hardened and rough dorsal skin with subcutaneous adhesion, and occasional scab formation. Mice in the Pred and Saff groups exhibited improvements in mental status, responsiveness, and behavior, along with reduced skin roughness, adhesions, and scabbing.

### Effects of safflower on fibrosis in skin, lung, and colon tissues

**FIGURE 2:**
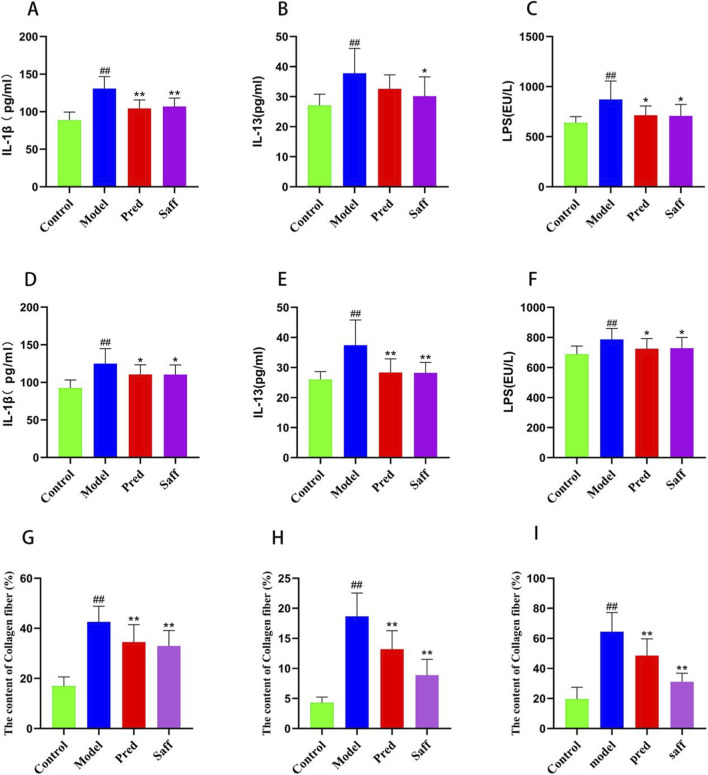
Biochemical indices in each group. Serum levels of **(A)** IL-1β, **(B)** IL-13, and **(C)** LPS, and colonic levels of **(D)** IL-1β, **(E)** IL-13, and **(F)** LPS. Collagen content in **(G)** skin tissue, **(H)** lung tissue, and **(I)** colon tissue. Data are presented as mean ± SD. ^##^P < 0.01 compared with the Control group; *P < 0.05 and **P < 0.01 compared with the Model group.

As shown in Figures 1A,E (HE and Masson’s trichrome staining), colonic tissue from the Control group displayed normal histology, with intact mucosal and glandular architecture, no inflammatory cell infiltration, and no collagen fiber accumulation. In the Model group, the epidermis and dermis of the skin were significantly thickened, with atrophy of other dermal structures, marked inflammatory infiltration, and increased collagen fiber deposition. Lung tissues showed prominent fibrosis and inflammatory infiltration, while colonic mucosa displayed epithelial damage, glandular disorganization, and pronounced inflammation within the lamina propria. In the Saff group, skin, lung, and colon tissue structures improved significantly. Collagen fiber deposition (blue-stained regions) was reduced and more uniformly distributed. These findings indicate that safflower treatment reduced collagen accumulation and fibrosis and alleviated inflammation in affected tissues. As shown in Figures 1B–D, semi-quantitative histopathological inflammation scoring demonstrated significantly increased inflammatory severity in the skin, lung, and colon of the model group compared with controls (P < 0.01). Consistently, Figures 1F–H indicated that quantitative assessment of fibrosis using collagen volume fraction (CVF) revealed markedly increased collagen deposition in the same tissues of the model group (P < 0.01), confirming the successful establishment of the fibrosis model. These pathological changes were significantly alleviated in the safflower group, as evidenced by significantly reduced inflammation scores and CVF values compared with the model group (P < 0.05, P < 0.01, or P < 0.0001, as indicated in the figures). Figures 2A–I demonstrate that, relative to Controls, the Model group showed significant collagen accumulation in dermal, pulmonary and colonic tissues and increased concentrations of IL-1β, IL-13 and LPS in both serum and colon. Inflammatory mediators (IL-1β, IL-13, and LPS) in the Model group were increased by approximately 1.4–1.8-fold in both serum and colon tissues compared with the Control group. Compared with the Model group, safflower administration resulted in an approximate 20%–35% reduction in these elevated cytokine levels (P < 0.05 or P < 0.01). These findings indicate that safflower significantly attenuates both systemic and intestinal inflammatory responses in SSc mice.

### Fecal metabolomicjs analysis in mice

#### Principal component analysis (PCA)

Fecal samples from each group were subjected to UPLC–Q-Orbitrap–MS analysis in both positive and negative ionization modes.

**FIGURE 3:**
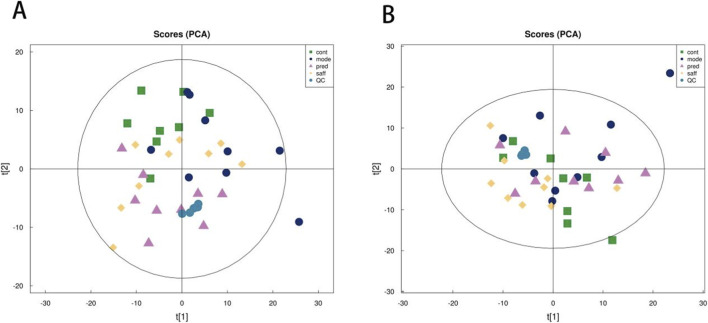
PCA score plots. **(A)** Positive ion mode; **(B)** Negative ion mode.

Principal component analysis (PCA) was performed on the original mass spectrometry dataset. As shown in Figure 3, across both ionization modes, the Model group was clearly separated from the Control group, indicating significant metabolic disruption caused by bleomycin and successful establishment of the SSc model. Moreover, after safflower intervention, the Saff group exhibited a distinct separation from the Model group, suggesting that safflower treatment significantly altered the metabolic profile in SSc mice.

### Identification of differential metabolites

**FIGURE 4:**
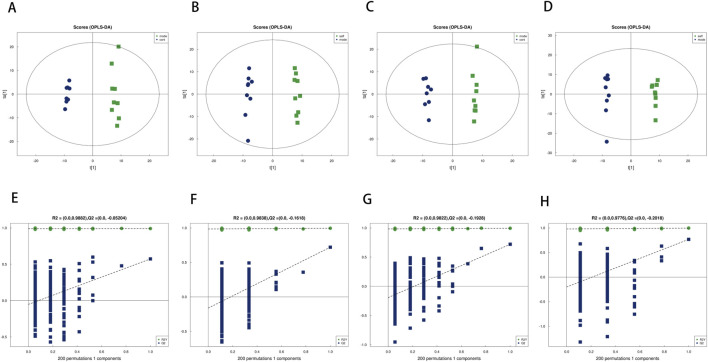
OPLS-DA score plots and permutation tests. **(A)** Model and Control groups in positive ion mode. **(B)** Saff and Model groups in positive ion mode. **(C)** Model and Control groups in negative ion mode. **(D)** Saff and Model groups in negative ion mode. **(E)** Permutation test for Model and Control groups in positive ion mode. **(F)** Permutation test for Saff and Model groups in positive ion mode. **(G)** Permutation test for Model and Control groups in negative ion mode. **(H)** Permutation test for Saff and Model groups in negative ion mode.

To further investigate the impact of safflower intervention on the fecal metabolic profile, orthogonal partial least squares-discriminant analysis (OPLS-DA) was applied to the endogenous metabolite data. As shown in the score plots (Figures 4A–H), clear separation was observed between the Control and Model groups, as well as between the Model and Saff groups, indicating a significant alteration in the metabolic state of model mice, which was ameliorated by safflower treatment. For the comparison between the Model and Saff groups, the OPLS-DA model demonstrated a high goodness of fit and predictive performance. In positive ion mode, the explained variance (R^2^Y) was 0.995 and the predictive parameter (Q^2^) was 0.720. In negative ion mode, the values were R^2^Y = 0.996 and Q^2^ = 0.767. The R^2^Y values close to 1 and Q^2^ values exceeding 0.5 in both modes indicated excellent model interpretability and high stability. The model incorporated 1,304 and 815 metabolic variables in the positive and negative ion modes, respectively. Model stability was assessed via 7-fold cross-validation. To ensure model validity and guard against overfitting, a permutation test with 200 iterations was performed following cross-validation. The permutation test results (Figures 4I,J) showed that the regression lines of *R* ^2^ and Q^2^ for the randomly permuted models intercepted the Y-axis below zero, and the Q^2^ values on the left were significantly lower than those of the original model on the right. This confirms that the original model possesses robust predictive capability without overfitting. Differential metabolites were screened based on a variable importance in projection (VIP) score >1.0 from the OPLS-DA model, combined with Student’s t-test (P < 0.05) and S-plot analysis. As listed in Table 2, a total of 26 differential metabolites were identified. Compared to the Control group, the Model group showed significantly decreased levels of 23 metabolites and increased levels of 3 metabolites (*P < 0.05, **P < 0.01). Notably, safflower intervention significantly reversed these aberrant metabolic changes compared to the Model group (*P < 0.05, **P < 0.01).

**TABLE 2:** Summary of differential fecal metabolites.

Functional category	Metabolite name	Adduct	Model vs. control				Saff vs. model			
			VIP score	Fold change (FC)	P-value	Change trend	VIP score	Fold change (FC)	P-value	Change trend
Amino acid-related metabolites	Aspartic acid	[M-H]+	3.515345625	3.217626287	0.043093951	↑	3.641609571	0.22568657	0.017270133	↓
	N-acetylcadaverine	[M + H]+	1.361624604	0.511938328	0.047290339	↓	2.460461176	3.57411347	0.037837493	↑
	D-mannosamine	[M + H]+	1.713866974	0.558372582	0.002361464	↓	1.585526592	1.559419621	0.014703436	↑
	N-acetyl-D-glucosamine 6-phosphate	[M-H]+	1.361624604	0.511938328	0.047290339	↓	2.460461176	3.57411347	0.037837493	↑
	4-pyridoxic acid	[M + H]+	2.349502338	0.759886934	0.037225393	↓	3.730214505	1.551221249	0.0160042	↑
	Kynurenic acid	[M-H]-	2.121939036	0.459607457	0.001905127	↓	2.323083122	2.555157654	0.00989723	↑
Lipid and phospholipid metabolites	1-myristoyl-2-hydroxy-sn-glycero-3-phosphoethanolamine	[M + H]+	2.458986336	0.509357335	0.009625982	↓	3.033251355	2.133842906	0.002052551	↑
	1-palmitoyl-2-hydroxy-sn-glycero-3-phosphoethanolamine	[M-H]-	3.843304425	0.530460294	0.00577572	↓	2.636932898	1.497594436	0.012744558	↑
	Leukotriene d4 methyl ester	[M + H]+	1.6427708	0.718536845	0.017907629	↓	1.888460905	1.456328171	0.023539257	↑
	Cis-9-palmitoleic acid	[M-H]-	1.25167983	0.31420042	0.009617383	↓	1.032129037	2.395332548	0.035869798	↑
	γ-heptalactone	[M + H]+	1.059641575	0.614295504	0.008753016	↓	1.275864945	1.658060873	0.008271845	↑
Steroid hormone-related metabolites	Androstendione	[M-H]-	5.274241498	0.364922431	0.000236505	↓	3.888608121	2.100412479	0.023992304	↑
	5alpha-androstane-3,17-dione	[M-H]-	2.416649639	3.137240204	0.005205371	↑	2.79909875	1.696684793	0.029960894	↑
	21-hydroxyprogesterone	[M-H]-	1.265342909	0.639485723	0.002819261	↓	1.342657965	1.644728702	0.001518124	↑
	Calcipotriol	[M + H]+	2.223415787	0.56312135	0.019638642	↓	3.040923863	2.050975787	0.004491213	↑
Phenolic and aromatic metabolites	2,6-dimethoxyphenol	[M + H]+	1.879383732	0.364623937	0.000642946	↓	1.793351374	2.242777025	0.000796028	↑
	4,6-diamino-5-formamidopyrimidine	[M + H]+	3.249812342	0.349047455	0.000500285	↓	2.210239237	1.884345657	0.019746946	↑
	Phillyrin	[M-H]-	1.374811629	0.509236337	0.010808397	↓	1.103427369	1.593303633	0.004621439	↑
	7-hydroxyflavanone	[M-H]-	2.244192172	0.192160618	0.024270886	↓	2.335404104	4.921747486	0.005237498	↑
	Quinoline-2,8-diol	[M-H]-	1.386282969	0.616642727	0.01345051	↓	1.680819024	1.842803312	0.005492258	↑
	2,5-dihydroxy-3,6-diphenyl-1,4-benzoquinone	[M-H]-	2.686984466	0.491625676	0.000233976	↓	2.135107101	1.771648738	0.01738033	↑
Other metabolites	Tubacin	[M + H]+	1.718766616	0.188807491	0.002357813	↓	1.716423227	5.017985409	0.02138755	↑
	Olopatadine	[M + H]+	1.071935571	1.827191677	0.011054235	↑	1.467259942	0.337357509	1.08542E-05	↓
	Protoporphyrin ix	[M + H]+	1.875827884	2.021443841	0.028322148	↑	2.050554023	0.352282776	0.004960115	↓
	Albocycline	[M-H]-	1.690276591	4.983458568	0.000420588	↑	2.292100862	3.06361494	0.048047849	↑
	4-[5-[[4-[5-[acetyl (hydroxy)amino] pentylamino]-4-oxobutanoyl] -hydroxyamino] pentylamino]-4-oxobutanoic acid	[M + H]+	2.111033297	0.578035728	0.007412893	↓	3.041346124	2.345859214	0.001385746	↑

#### KEGG pathway enrichment analysis

KEGG pathway enrichment analysis of 26 differential metabolites (P < 0.05) revealed significant enrichment in 18 metabolic pathways, including central carbon metabolism in cancer, aldosterone synthesis and secretion, alanine, aspartate, and glutamate metabolism, mineral absorption, protein digestion and absorption, aminoacyl-tRNA biosynthesis, and steroid hormone biosynthesis, as shown in Figure 9A.

### Gut microbiota analysis

#### Diversity analysis

Alpha diversity reflects within-community diversity (species richness and evenness) and is commonly quantified by indices including the Shannon and Simpson diversity measures, alongside richness estimators such as ACE and Chao1.

**FIGURE 5:**
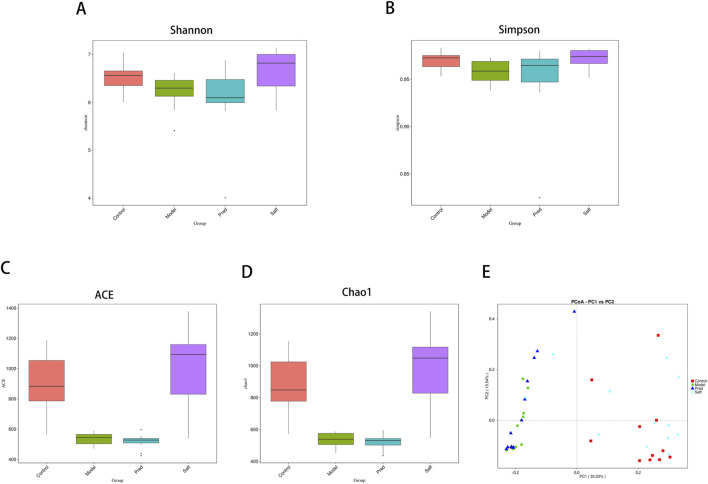
Alpha- and beta-diversity analyses of gut microbiota. **(A–D)** Intergroup comparisons of alpha-diversity indices, including Shannon, Simpson, ACE, and Chao1 indices. **(E)** Principal coordinate analysis (PCoA) plot of beta diversity based on unweighted UniFrac distances.

Figures 5A–D indicates that all alpha-diversity metrics in the Model group were lower than those in the Control cohort (P < 0.05 or P < 0.01), reflecting diminished gut microbial richness and evenness in SSc mice. Comparisons between the Model and Pred groups did not reach statistical significance (P > 0.05). Following safflower administration, Shannon, Simpson, ACE, and Chao1 indices exhibited marked increases (P < 0.01), suggesting that safflower helped restore intestinal microbial diversity in SSc mice.

Beta diversity was evaluated through the application of principal coordinate analysis (PCoA), utilizing unweighted UniFrac distances as the basis for the assessment. As shown in Figure 5E, fecal samples from the Model and Control groups showed clear separation in the PCoA plot, indicating distinct microbial community structures. After safflower treatment, the Saff group samples clustered closer to those of the Control group, with partial overlap, suggesting that safflower treatment ameliorated gut microbiota dysbiosis and promoted restoration of microbial community composition in SSc mice.

#### Composition and differential analysis of gut microbiota

**FIGURE 6:**
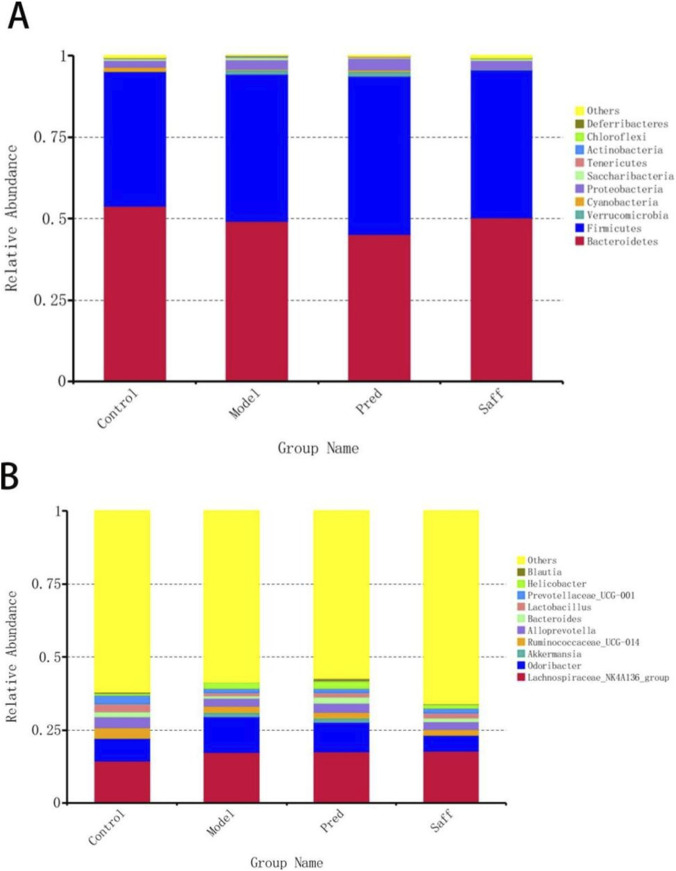
Microbial composition at the phylum and genus levels. **(A)** Relative abundance of microbial taxa at the phylum level. **(B)** Relative abundance of microbial taxa at the genus level.

**FIGURE 7:**
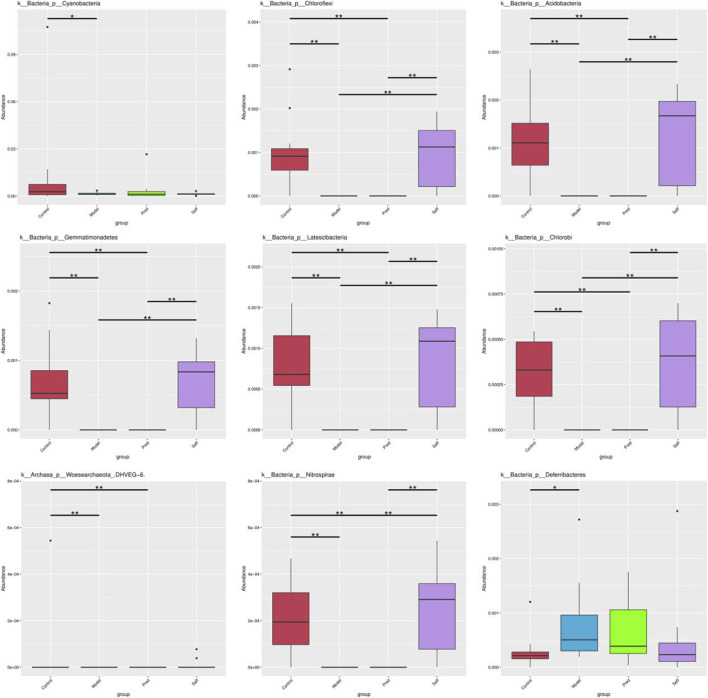
Comparison of the relative abundances of differential phyla among groups. Data are presented as mean ± SD.

At the phylum level, the gut microbiota was mainly composed of Bacteroidetes and Firmicutes (Figure 6A). The Model group showed significant microbial imbalance compared to the Control group, with reduced low-abundance phyla like *Cyanobacteria*, *Chloroflexi*, and *Acidobacteria*, and increased *Deferribacteres* (P < 0.05). Safflower treatment partially reversed this imbalance, notably restoring *Chloroflexi* and *Acidobacteria* levels, potentially affecting fibrosis or immune regulation (P < 0.05, Figure 7).

**FIGURE 8:**
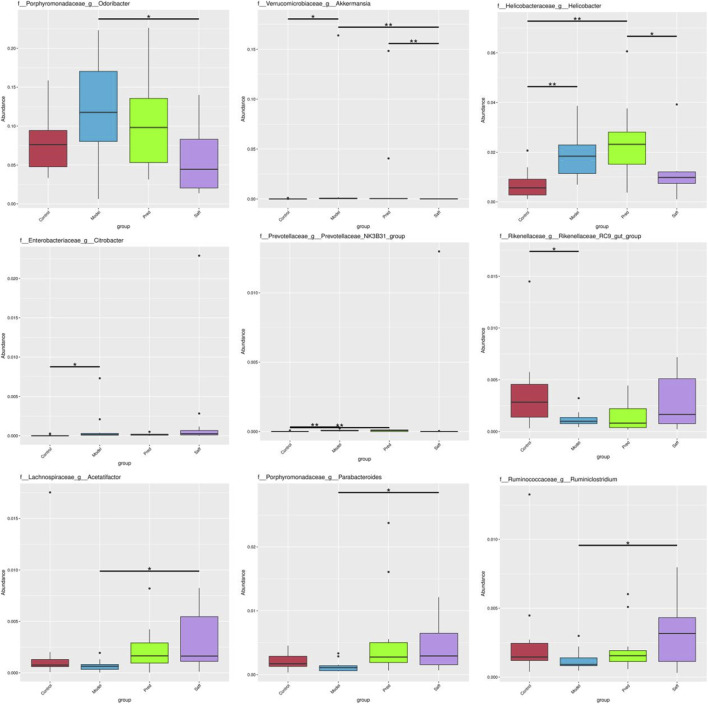
Comparison of the relative abundances of differential genera among groups. Data are presented as mean ± SD.

At the genus level, the gut microbiota composition changed significantly after model induction, with increases in inflammation- and fibrosis-associated genera like Odoribacter and decreases in beneficial short-chain fatty acid producers (Figure 6B). Safflower intervention reversed these changes, reducing the pro-fibrotic genus Odoribacter and increasing protective genera like Acetatifactor (P < 0.05). Figure 8 summarizes the most significant and relevant bacterial genera.

#### Correlation analysis between gut microbiota and differential fecal metabolites

Spearman’s rank correlation was performed to explore functional associations between differential gut microbiota and the fecal metabolome in SSc mice. To reduce statistical noise, only moderate to strong correlations (|ρ| ≥0.5, P < 0.05) were considered biologically meaningful and emphasized.

**FIGURE 9:**
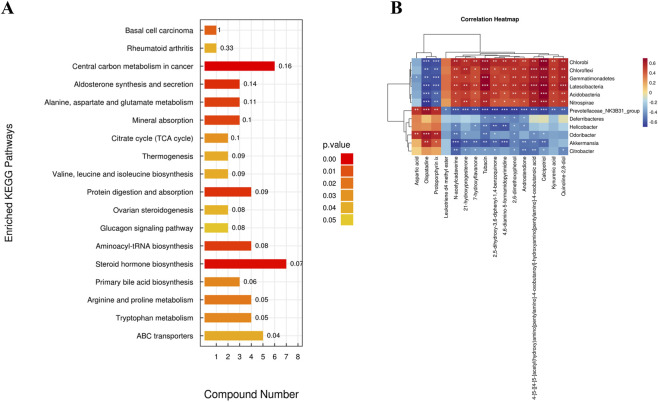
Correlation analysis between differential metabolites and gut microbiota. **(A)** KEGG pathway enrichment analysis of differential metabolites. **(B)** Spearman correlation analysis between differential metabolites and gut microbiota.

As shown in Figure 9B, multiple associations were shown to exist between particular metabolites and microbiological taxa: Aspartic acid and Protoporphyrin IX were positively correlated with Prevotellaceae*_NK3B31_group*, *Odoribacter*, and *Akkermansia*, but negatively correlated with *Chloroflexi*, *Acidobacteria*, *Gemmatimonadetes*, *Chlorobi*, and *Nitrospirae*. Kynurenic acid demonstrated a favorable association with *Chloroflexi*, *Acidobacteria*, *Gemmatimonadetes*, *Chlorobi*, and *Nitrospirae*, and a negative correlation with Prevotellaceae*_NK3B31_group*. Prevotellaceae*_NK3B31_group* had a strong negative correlation with the methyl ester of Leukotriene D4. Our results indicate that safflower may attenuate inflammation and fibrosis in SSc mice by altering gut microbiota composition and consequently modulating specific metabolic networks.

Collectively, these findings suggest that safflower treatment may attenuate inflammation and fibrosis in SSc mice by reshaping gut microbiota composition and subsequently modulating specific metabolic networks.

## Discussion

Scleroderma is an autoimmune disease characterized by excessive collagen deposition and diffuse skin and internal organ fibrosis. Establishing a reliable animal model of scleroderma is fundamental for researching its etiology, pathogenesis, and for evaluating the efficacy of therapeutic interventions. Among available models, the bleomycin (BLM)-induced SSc model, first described by Yamamoto et al. (Yamamoto et al., 1999), has been widely used in basic research owing to its close resemblance to human SSc in terms of skin fibrosis, systemic inflammation, and autoimmune responses (Beyer et al., 2010). In this study, we employed the BLM-induced SSc model and assessed safflower’s efficacy using clinical symptoms, histopathology, and biochemical markers. We found that safflower treatment significantly improved skin sclerosis, reduced collagen content in skin, lung, and colon tissues, alleviated fibrosis and inflammatory cell infiltration, and decreased serum and colonic levels of IL-1β, IL-13, and LPS. We further explored the potential mechanisms of the therapeutic effect of safflower. Metabolomic analysis revealed that safflower treatment can regulate the disturbances in amino acid, lipid, and energy metabolism in the Model group, shifting the overall metabolic profile toward that of the Control group. Pathway enrichment analysis indicated that the differential metabolites were mainly enriched in tryptophan metabolism as well as alanine, aspartate, and glutamate metabolism. Concurrently, safflower intervention can also improve the disturbance of gut microbiota dysbiosis in SSc mice, increase the relative abundance of beneficial bacterial taxa, such as *Chloroflexi* and *Acidobacteria*, and reduce the abundance of potentially pathogenic or pro-inflammatory genera, including *Odoribacter* and *Akkermansia*. The following text will further explore the potential mechanism of safflower in improving systemic sclerosis from three aspects: changes in metabolic pathways, imbalance of gut microbiota, and their interaction.

Kynurenic acid (KYNA) is a critical product of the kynurenine pathway (KP), which metabolizes tryptophan (TRP) (Turska-Kozłowska et al., 2024). Under inflammatory conditions, cytokines such as IFN-γ, IL-1, IL-6, and TNF-α stimulate KP activation, increasing KYNA production (Wirthgen et al., 2018). KYNA promotes regulatory T cell differentiation and inhibits pro-inflammatory cytokines by activating the aryl hydrocarbon receptor (AhR) and modulating pathways such as NF-κB and GPR35 (Stone et al., 2024; Wang et al., 2022). In patients with SSc, TRP levels are reduced, while the ratios of KYNA/TRP and KYN/TRP are elevated (Turska-Kozłowska et al., 2024). Aspartic acid, a non-essential amino acid, is vital for leukocyte metabolism and nucleotide synthesis, playing a crucial part in lymphocyte proliferation (Li et al., 2007). Studies show decreased aspartic acid levels in the serum of SSc patients, and a positive correlation between aspartic acid levels and the Modified Rodnan Skin Score (mRSS), a clinical marker of skin involvement in SSc (Murgia et al., 2018; Jud et al., 2023). Arachidonic acid (AA) is a polyunsaturated fatty acid that exists in cell membranes as a phospholipid. When inflammation or cell activation occurs, cPLA2 in the cytoplasm hydrolyzes membrane-bound AA into a free form (Wang et al., 2021). Then, 5-LOX oxidizes free AA into 5-HPETE, which is further converted into LTD4 by a series of enzymes (Liu and Ai, 2025). As a potent inflammatory mediator, LTD4 activates CysLT receptors and works with cytokines like IL-5 and GM-CSF to induce TGF-β expression and proteoglycan synthesis. This promotes eosinophil activation, intensifying inflammation and fibrosis (Punjasamanvong and Muangchan, 2021). LTD4 is involved in various inflammatory diseases and cancers (Cabral et al., 2015). Studies have shown that serum LTD4 levels are significantly higher in scleroderma patients than in healthy people (Mandujano et al., 2021). Heme and downstream metabolites may contribute to the pathogenesis of scleroderma. Clinical studies have reported that vascular endothelial cell expression of heme oxygenase-1 (HO-1) is significantly decreased in patients with scleroderma relative to healthy individuals. The downregulation of HO-1 may lead to a decrease in the antioxidant capacity of vascular endothelial cells, thereby exacerbating vascular lesions in patients with scleroderma (Ross et al., 2022). The platelet-to-lymphocyte ratio (PLR) and platelet-to-hemoglobin ratio (PHR) were found to be significantly associated with disease activity and clinical severity in scleroderma. PLR and PHR were positively associated with inflammatory markers—erythrocyte sedimentation rate (ESR) and C-reactive protein (CRP)—as well as with the scleroderma activity index (EScSG-AI) (Sarhan and El-Meligui, 2023). This suggests that heme and its metabolites may be involved in the inflammatory process of scleroderma. Protoporphyrin IX (PPIX) is an important intermediate in the heme biosynthesis pathway. It is catalyzed by ferrochelatase in the mitochondria to bind with ferrous ions and be converted into heme. A decrease in PPIX levels can lead to impaired heme synthesis (Sachar et al., 2016). These studies indicate that the pathogenesis of systemic sclerosis is closely associated with metabolic disturbances involving tryptophan, aspartate, LTD4, and PPIX. In the present study, we observed increased fecal levels of aspartate and PPIX, along with decreased levels of kynurenic acid and leukotriene D4 methyl ester in SSc mice, whereas safflower treatment effectively reversed these metabolic alterations. These changes may represent potential mechanisms underlying the inhibitory effects of safflower on pro-inflammatory mediator production and its anti-fibrotic activity. Notably, our metabolomic analysis based on fecal samples revealed metabolic trends that differed from those reported in previous studies using serum or plasma samples. We speculate that these discrepancies may be related to differences in inflammatory cytokine profiles between systemic circulation and the intestinal milieu, as well as to the distinct pro-inflammatory or anti-inflammatory roles of specific metabolites. However, the precise mechanisms underlying these differences remain unclear and warrant further investigation.

16S rRNA sequencing revealed that the gut microbiota structure of SSc mice was significantly altered compared with that of the Control group, with pronounced changes observed across taxonomic levels from phylum to genus. Deferribacteres phylum, considered a conditionally pathogenic taxon, has been associated with intestinal barrier dysfunction and inflammatory status. Studies have found that its abundance is increased in mice with inflammatory bowel disease (Han et al., 2021). *Chloroflexi* phylum is a beneficial bacterium that can maintain protein stability in high-temperature environments to sustain cellular life activities (Narsing Rao et al., 2022), and its abundance is reduced in patients with rheumatoid arthritis (Mao et al., 2020). Other studies have reported that reduced abundances of *Cyanobacteria*, *Acidobacteria*, *Gemmatimonadetes* and *Nitrospirae* are associated with impaired growth performance in piglets, potentially due to elevated oxidative stress and intensified inflammation (Li et al., 2019; Li et al., 2020). An appropriate level of *Odoribacter* can promote the generation and differentiation of peripheral Treg cells, inhibit inflammatory factors, affect immune regulation, and protect the intestinal mucosa, but excessive levels may be pathogenic (Liu Y. et al., 2021). Studies have reported elevated relative abundances of *Odoribacter* during exacerbations of allergic rhinitis in children and in murine models of colon cancer; *Odoribacter* may modulate immune inflammation and tumor progression by producing pro-inflammatory mediators (Lin et al., 2020). *Helicobacter* can mediate inflammation and immune responses to induce disease, and its subordinates can induce collagen vascular disease-related interstitial pneumonia and idiopathic pulmonary fibrosis (Nakashima et al., 2015). A decrease in *Akkermansia* in the gut can lead to weakened intestinal barriers, bacterial toxin translocation, and the development of inflammatory bowel disease and type 2 diabetes (Hasani et al., 2021; Zhang et al., 2021); conversely, it may “activate” the activation threshold of immune cells, leading to excessive immune responses (Bae et al., 2022). *Lactobacillus* is a well-known beneficial genus with anti-inflammatory and immune response-lowering effects. According to the latest research (Aghamohammad et al., 2022), *Lactobacillus* can inhibit inflammation in HT-29 cells by regulating the JAK/STAT and NF-kB pathways, reducing the production of IL-6 and IL-1β, and preventing and lowering the prevalence of illnesses linked to inflammation, such as IBD. Prevotella_UCG-001 is a short-chain fatty acid (SCFA)-producing bacterium (Tett et al., 2021), and its abundance is positively correlated with anti-inflammatory effects (Jialing et al., 2020). Lipopolysaccharide (LPS) is an endotoxin that can cause a strong immune response and promote the secretion of cytokines by various cells. LPS from Gram-negative bacteria in the gut microbiota is key to initiating systemic chronic inflammatory responses, and it can promote the binding of CD14 and TLR-4, transmit immune signals from CD14 and TLR4, and release inflammatory factors (Gu et al., 2017). Moreover, LPS can activate TLR-4 in the mononuclear phagocyte system to produce a large number of fibrotic cytokines (TGF-β1, IL-2), which directly or indirectly act on tissue fibroblasts, promoting their expression of α-SMA and proliferation and activation into myofibroblasts, synthesizing and secreting collagen, and ultimately leading to organ fibrosis (Olman et al., 2004). An increase in Gram-negative bacteria can promote the synthesis of LPS in the cell wall, accelerating the inflammatory response process (Luo and Yang, 2018). Inflammatory factors caused by intestinal microbiota dysregulation play a key role in mediating organ fibrosis. Gasparini et al. (Gasparini et al., 2020) found that IL-13 promotes SSc inflammatory fibrosis by activating the differentiation of fibroblasts into collagen and maintains the fibrotic state of affected organs. The relative abundance of some bacterial genera is consistent with changes in specific cytokine levels, with the abundance of *Deferribacteres* (Leon et al., 2020; Liu S. et al., 2019), *Helicobacter* (El Filaly et al., 2023), and Odoribacter (Lin et al., 2019; Xing et al., 2021) being positively correlated with IL-1 and IL-13 levels. *Akkermansia* is associated with Th1 polarization of T cells, and the higher its abundance, the higher the level of pro-inflammatory cytokine IL-1β (Van Averbeke et al., 2022). In the Model group, proportions of *Deferribacteres*, *Odoribacter*, *Akkermansia* and *Helicobacter* were significantly elevated, implying increased LPS levels in bleomycin (BLM)-induced SSc mice that induced fibrotic and inflammatory responses in skin, lung and colon tissues—findings consistent with histopathology and physiological/biochemical indices. After safflower treatment, the symptoms and corresponding indicators of SSc mice were improved, the abundance of *Chloroflexi*, *Acidobacteria*, *Gemmatimonadetes*, *Latescibacteria*, *Chlorobi*, *Nitrospirae*, *Acetatifactor*, *Parabacteroides*, and *Ruminiclostridium* was upregulated, and the abundance of *Odoribacter*, *Akkermansia*, and *Helicobacter* was downregulated. In summary, safflower may suppress pathogenic bacteria while enriching beneficial commensal populations, thereby attenuating inflammatory progression and mitigating tissue fibrosis.

In this study, Spearman’s rank correlation was applied to assess the associations between differential fecal metabolites and gut microbiota. The results showed that the metabolites Aspartic acid and Protoporphyrin IX were elevated in the model group, while Kynurenic acid was decreased. Aspartic acid, a non-essential amino acid, participates in amino acid metabolism, the tricarboxylic acid (TCA) cycle, the urea cycle, and neurotransmitter synthesis (Holeček, 2023). Aspartic acid has been proven to be significantly reduced in the serum of SSc patients and is closely related to the degree of skin sclerosis (Murgia et al., 2018; Jud et al., 2023). Overaccumulation of Protoporphyrin IX in the body can cause severe phototoxic reactions, leading to lipid peroxidation of cell membranes and activation of complement cells, mast cells, and TGF-β to initiate the fibrosis process, causing skin sclerosis (Romanowska-Próchnicka et al., 2024). Kynurenic acid and its metabolites can inhibit the metabolism and proliferation of fibroblasts, thereby reducing fibrosis (Turska-Kozłowska et al., 2024). *Chloroflexi*, *Acidobacteria*, *Gemmatimonadetes*, *Chlorobi*, and *Nitrospirae* are all beneficial bacterial genera that can regulate immune responses by producing anti-inflammatory cytokines and are involved in the pathogenesis of various autoimmune diseases, including rheumatoid arthritis (Dahal et al., 2023). According to the Spearman correlation coefficient, *Chloroflexi*, *Acidobacteria*, *Gemmatimonadetes*, *Chlorobi*, and *Nitrospirae* were positively associated with kynurenic acid and inversely associated with aspartic acid and protoporphyrin IX. Safflower supplementation enhanced the representation of *Chloroflexi*, *Acidobacteria*, *Gemmatimonadetes*, *Chlorobi*, and *Nitrospirae* within the gut microbiota. Therefore, elevated proportions of *Chloroflexi*, together with *Acidobacteria*, *Gemmatimonadetes*, *Chlorobi*, and *Nitrospirae*, may ameliorate inflammation and fibrosis in SSc through modulation of aspartate, heme, and tryptophan metabolic pathways. This reports the correlation between fecal metabolites and gut microbiota.

The present study provides preliminary evidence suggesting that safflower may ameliorate systemic sclerosis–associated fibrotic and inflammatory phenotypes through modulation of the “gut microbiota–host metabolism” axis; however, several important limitations should be acknowledged. First, only a single dose of safflower aqueous extract was evaluated in the present study, which precludes assessment of dose–response relationships or determination of an optimal pharmacological dose. This dose was selected based on preliminary dose-screening experiments conducted in the same experimental model, together with reference to dose ranges reported in prior peer-reviewed animal studies, with the primary aim of detecting an initial biological signal rather than establishing definitive pharmacodynamic parameters. Accordingly, the present study should be regarded as exploratory and hypothesis-generating in nature. Second, although associations between gut microbiota alterations, fecal metabolic changes, and fibrotic phenotypes were identified using 16S rRNA sequencing and untargeted metabolomics, no targeted *in vivo* or *in vitro* experiments were performed to validate specific molecular targets or signaling pathways. In particular, the absence of cell-based assays limits inference regarding upstream or downstream regulatory mechanisms. Therefore, the proposed microbiota–metabolism–host interactions should be interpreted as correlative rather than causal. Third, the mechanisms underlying the discrepant metabolic trends between fecal and serum samples, as well as the causal contribution of gut microbiota remodeling to therapeutic efficacy, remain to be clarified. Future studies incorporating targeted pathway analyses, cell-based assays, and microbiota manipulation approaches (e.g., fecal microbiota transplantation) are warranted to establish causality and validate key mechanistic targets. Fourth, it is not yet clear which specific bacterial taxa or their functional metabolites play dominant roles in driving metabolic regulation. Future studies should integrate multi-compartment metabolomic analyses, including serum and tissue samples, to comprehensively characterize metabolite dynamics and to verify the necessity of gut microbiota in mediating the pharmacological effects of safflower. Such approaches will facilitate a more precise elucidation of the molecular mechanisms underlying the “microbiota-metabolism-host” interactions involved in systemic sclerosis.

It should be emphasized that the present study was designed as an early exploratory investigation aimed at preliminarily evaluating the effects of an aqueous extract of safflower, a botanical drug listed in the pharmacopoeia, on fibrotic and inflammatory phenotypes in a murine model of systemic sclerosis. In the present study, animals were administered a standardized liquid aqueous extract of safflower prepared under controlled conditions. Based on the experimentally determined extraction yield (24.7%, w/w), the working concentration of the dosing suspension (37.1 mg/mL of freeze-dried extract) and a fixed gavage volume of 10 mL/kg, the accurate daily dose administered to mice was 371 mg of freeze-dried extract per kg body weight per day. The crude botanical drug equivalent value (1.5 g/kg/day) is provided solely as a reference unit and does not represent the actual amount of solid extract administered to the animals. We acknowledge that high-dose botanical preparations may be associated with non-specific effects and a potential risk of experimental artifacts. Consistent with the exploratory design and limited sample size, formal multiple-testing correction and complex multivariable regression modeling were not applied, as these approaches may increase the risk of overfitting. Similarly, formal confidence interval estimation was not performed, as the study was not designed for confirmatory inferential modeling. Instead, findings were interpreted in conjunction with effect size, biological plausibility, and consistency across tissues, and should therefore be regarded as hypothesis-generating preliminary signals rather than definitive pharmacodynamic conclusions. Nevertheless, the consistent improvements observed in fibrosis severity and inflammatory responses across skin, lung, and colon tissues following safflower intervention provide a reasonable rationale for further investigation.

In accordance with the principles of responsible pharmacological research (3R/4R principles), this study was designed to minimize experimental animal use while enabling the acquisition of preliminary efficacy and mechanistic insights. Pharmacokinetic profiling, the identification and functional validation of key plant-derived metabolites, systematic safety and toxicity evaluation, multi-dose validation, and functional outcome assessments (e.g., respiratory function tests or lung compliance testing) will be essential components of future confirmatory investigations aimed at defining the minimal effective dose, elucidating the molecular basis of safflower’s anti-fibrotic effects, and establishing its safe exposure range and translational relevance in systemic sclerosis.

## Conclusion

The present study indicates that safflower ameliorates fibrotic and inflammatory phenotypes in a murine model of systemic sclerosis. These effects appear to involve coordinated regulation of the “gut microbiota–host metabolism” axis. Safflower treatment reshaped the intestinal microbial ecosystem by increasing beneficial taxa, such as Acetatifactor, while reducing potentially pathogenic genera, including Odoribacter, and concurrently corrected disturbances in key metabolic pathways related to tryptophan, aspartate, porphyrin, and leukotriene D4 (LTD4) metabolism. Importantly, restoration of beneficial gut microbiota was significantly associated with changes in protective metabolites, suggesting that the therapeutic effects of safflower may be mediated through microbiota remodeling and subsequent systemic metabolic regulation. Collectively, these findings provide preliminary evidence supporting a gut axis–oriented strategy for modulating autoimmune fibrotic diseases.

## Data Availability

The raw sequencing data generated in this study is deposited in the NCBI Sequence Read Archive (SRA) under the BioProject accession number PRJNA1415796. The untargeted metabolomics raw data has been deposited in the National Genomics Data Center (NGDC, China National Center for Bioinformation; https://ngdc.cncb.ac.cn/omix/) under the accession number OMIX015049.

## References

[B1] Aghamohammad S. Sepehr A. Miri S. T. Najafi S. Pourshafie M. R. Rohani M. (2022). Anti-inflammatory and immunomodulatory effects of lactobacillus spp. as a preservative and therapeutic agent for IBD control. Immun. Inflamm. Dis. 10 (6), e635. 10.1002/iid3.635 35634951 PMC9119005

[B2] Allanore Y. Simms R. Distler O. Trojanowska M. Pope J. Denton C. P. (2015). Systemic sclerosis. Nat. Rev. Dis. Prim. 1 (0), 15002. 10.1038/nrdp.2015.2 27189141

[B3] Bae M. Cassilly C. D. Liu X. Park S. M. Tusi B. K. Chen X. (2022). Akkermansia muciniphila phospholipid induces homeostatic immune responses. Nature 608 (7921), 168–173. 10.1038/s41586-022-04985-7 35896748 PMC9328018

[B4] Bai H. Yang J. Wang R. (2025). Carthamus tinctorius L.: a comprehensive review of its ethnomedicine, phytochemistry, pharmacology, and clinical applications. Front. Pharmacol. 16, 1609299. 10.3389/fphar.2025.1609299 40918514 PMC12408597

[B5] Bairkdar M. Rossides M. Westerlind H. Hesselstrand R. Arkema E. V. Holmqvist M. (2021). Incidence and prevalence of systemic sclerosis globally: a comprehensive systematic review and meta-analysis. Rheumatol. Oxf. Engl. 60 (7), 3121–3133. 10.1093/rheumatology/keab190 33630060 PMC8516513

[B6] Bellando-Randone S. Russo E. Di Gloria L. Lepri G. Baldi S. Fioretto B. S. (2024). Gut microbiota in very early systemic sclerosis: the first case-control taxonomic and functional characterisation highlighting an altered butyric acid profile. RMD Open 10 (4), e004647. 10.1136/rmdopen-2024-004647 39557490 PMC11574430

[B7] Beyer C. Schett G. Distler O. Distler J. H. (2010). Animal models of systemic sclerosis: prospects and limitations. Arthritis Rheumatism 62 (10), 2831–2844. 10.1002/art.27647 20617524

[B8] Bhosle A. Wang Y. Franzosa E. A. Huttenhower C. (2022). Progress and opportunities in microbial community metabolomics. Curr. Opin. Microbiol. 70, 102195. 10.1016/j.mib.2022.102195 36063685

[B9] Cabral M. Martín-Venegas R. Moreno J. J. (2015). Leukotriene D4-induced Caco-2 cell proliferation is mediated by prostaglandin E2 synthesis. Physiol. Rep. 3 (7), e12417. 10.14814/phy2.12417 26216432 PMC4552517

[B10] Chen P. Y. (2007). Decoction methods for optimizing extraction yield in Chinese herbal medicine. Med. J. Chin. People' S Health. (12), 496–497.

[B11] Chen L. Chen H. Chen Z. Zhang K. Zhang H. Xu J. (2025). Shared pathogenic mechanisms linking obesity and idiopathic pulmonary fibrosis revealed by bioinformatics and *in vivo* validation. Sci. Rep. 15 (1), 25896. 10.1038/s41598-025-12046-y 40670650 PMC12267603

[B12] Cossu M. van Bon L. Preti C. Rossato M. Beretta L. Radstake T. R. D. J. (2017). Earliest phase of systemic sclerosis typified by increased levels of inflammatory proteins in the serum. Arthritis and Rheumatology (Hoboken, N.J.). 69 (12), 2359–2369. 10.1002/art.40243 28859262

[B13] Cui D. Zhao D. Huang S. (2020). Structural characterization of a safflower polysaccharide and its promotion effect on steroid-induced osteonecrosis *in vivo* . Carbohydr. Polym. 233, 115856. 10.1016/j.carbpol.2020.115856 32059907

[B14] Dahal R. H. Kim S. Kim Y. K. Kim E. S. Kim J. (2023). Insight into gut dysbiosis of patients with inflammatory bowel disease and ischemic colitis. Front. Microbiology 14, 1174832. 10.3389/fmicb.2023.1174832 37250025 PMC10211348

[B15] Denton C. P. Khanna D. (2017). Systemic sclerosis. Lancet London, Engl. 390 (10103), 1685–1699. 10.1016/S0140-6736(17)30933-9 28413064

[B16] El Filaly H. Outlioua A. Desterke C. Echarki Z. Badre W. Rabhi M. (2023). IL-1 polymorphism and *Helicobacter pylori* infection features: highlighting VNTR's potential in predicting the susceptibility to infection-associated disease development. Microorganisms 11 (2), 353. 10.3390/microorganisms11020353 36838318 PMC9961292

[B17] Fu H. Liu X. Jin L. Lang J. Hu Z. Mao W. (2021). Safflower yellow reduces DEN-induced hepatocellular carcinoma by enhancing liver immune infiltration through promotion of collagen degradation and modulation of gut microbiota. Food and Funct. 12 (21), 10632–10643. 10.1039/d1fo01321a 34585698

[B18] Gasparini G. Cozzani E. Parodi A. (2020). Interleukin-4 and interleukin-13 as possible therapeutic targets in systemic sclerosis. Cytokine 125, 154799. 10.1016/j.cyto.2019.154799 31400638

[B19] Gu N. N. Zhang X. D. Yu H. L. Xie H. Zhu X. X. Li Y. (2017). Based on sequencing of 16S rRNA gene elaborate effect of Coptis chinensis on intestinal microbial diversity in type 2 diabetes mellitus. Chin. Traditional Herb. Drugs 48 (19), 3998–4004.

[B20] Guo L. S. Jia Y. L. (1996). A study on the decoction methods of Chinese herbal medicine. Chin. Tradit. Pat. Med. (08), 8–9.

[B21] Han S. K. Shin Y. J. Lee D. Y. Kim K. M. Yang S. J. Kim D. S. (2021). Lactobacillus rhamnosus HDB1258 modulates gut microbiota-mediated immune response in mice with or without lipopolysaccharide-induced systemic inflammation. BMC Microbiol. 21 (1), 146. 10.1186/s12866-021-02192-4 33985438 PMC8120827

[B22] Hasani A. Ebrahimzadeh S. Hemmati F. Khabbaz A. Hasani A. Gholizadeh P. (2021). The role of Akkermansia muciniphila in obesity, diabetes and atherosclerosis. J. Med. Microbiol. 70 (10). 10.1099/jmm.0.001435 34623232

[B23] Hevia A. Milani C. López P. Cuervo A. Arboleya S. Duranti S. (2014). Intestinal dysbiosis associated with systemic lupus erythematosus. mBio 5 (5), e01548. 10.1128/mBio.01548-14 25271284 PMC4196225

[B24] Holeček M. (2023). Aspartic acid in health and disease. Nutrients 15 (18), 4023. 10.3390/nu15184023 37764806 PMC10536334

[B25] Hu M. B. Lü J. Y. (2018). Effect of safflower water extract on treg CellRelated factors in mice with systemic sclerosis. J. Hubei Univ. Nationalities. Medical Ed. 35 (04), 5–9. 10.13501/j.cnki.42-1590/r.2018.04.002

[B26] Jialing L. Yangyang G. Jing Z. Xiaoyi T. Ping W. Liwei S. (2020). Changes in serum inflammatory cytokine levels and intestinal flora in a self-healing dextran sodium sulfate-induced ulcerative colitis murine model. Life Sci. 263, 118587. 10.1016/j.lfs.2020.118587 33065145

[B27] Jud P. Meinitzer A. Strohmaier H. Arefnia B. Wimmer G. Obermayer-Pietsch B. (2023). Association of amino acids and parameters of bone metabolism with endothelial dysfunction and vasculopathic changes in limited systemic sclerosis. Front. Med. 10, 1193121. 10.3389/fmed.2023.1193121 37425312 PMC10327605

[B28] Klingberg E. Magnusson M. K. Strid H. Deminger A. Ståhl A. Sundin J. (2019). A distinct gut microbiota composition in patients with ankylosing spondylitis is associated with increased levels of fecal calprotectin. Arthritis Res. and Ther. 21 (1), 248. 10.1186/s13075-019-2018-4 31771630 PMC6880506

[B29] Leon I. C. Quesada-Vázquez S. Sáinz N. Guruceaga E. Escoté X. Moreno-Aliaga M. J. (2020). Effects of maresin 1 (MaR1) on colonic inflammation and gut dysbiosis in diet-induced Obese mice. Microorganisms 8 (8), 1156. 10.3390/microorganisms8081156 32751593 PMC7465372

[B30] LeRoy E. C. Black C. Fleischmajer R. Jablonska S. Krieg T. Medsger T. A. (1988). Scleroderma (systemic sclerosis): classification, subsets and pathogenesis. J. Rheumatology 15 (2), 202–205. 3361530

[B31] Li P. Yin Y. L. Li D. Kim S. W. Wu G. (2007). Amino acids and immune function. Br. J. Nutr. 98 (2), 237–252. 10.1017/S000711450769936X 17403271

[B32] Li Y. Han H. Yin J. He X. Tang Z. Li T. (2019). d-and l-Aspartate regulates growth performance, inflammation and intestinal microbial community in young pigs. Food and Funct. 10 (2), 1028–1037. 10.1039/c8fo01410h 30706916

[B33] Li Y. Wang P. Yin J. Jin S. Su W. Tian J. (2020). Effects of ornithine α-ketoglutarate on growth performance and gut microbiota in a chronic oxidative stress pig model induced by d-galactose. Food and Funct. 11 (1), 472–482. 10.1039/c9fo02043h 31833510

[B34] Li Y. Q. Huang Y. Zhou X. Y. Zhao L. J. Lyu J. Y. (2022). Effect of safflower water extract on skin and pulmonary fibrosis in bleomycin-induced systemic scleroderma mice. Intern. Med. 17 (02), 134–140. 10.16121/j.cnki.cn45-1347/r.2022.02.03

[B35] Lin C. H. Chen C. C. Chiang H. L. Liou J. M. Chang C. M. Lu T. P. (2019). Altered gut microbiota and inflammatory cytokine responses in patients with parkinson's disease. J. Neuroinflammation 16 (1), 129. 10.1186/s12974-019-1528-y 31248424 PMC6598278

[B36] Lin Y. H. Rui X. Q. Li Y. J. (2020). Role of gut microbiota in children with allergic rhinitis with high serum total IgE level. J. Clin. Otorhinolaryngology Head Neck Surg. 34 (12), 1123–1128. 10.13201/j.issn.2096-7993.2020.12.016 33254349 PMC10127780

[B37] Liu T. Ai D. (2025). Roles of lipoxygenases in cardiovascular diseases. J. Cardiovasc. Transl. Res. 18 (3), 599–610. 10.1007/s12265-025-10605-2 40133736 PMC12209014

[B38] Liu Y. Yu X. J. Yu L. L. Tian F. W. Zhao J. X. Zhang H. (2021). Lactobacillus plantarum CCFM8610 alleviates irritable bowel syndrome and prevents gut microbiota dysbiosis: a Randomized,Double-Blind, placebo-controlled, pilot clinical trial. Engineering 7 (03), 226–247. 10.1016/j.eng.2020.06.026

[B39] Liu J. J. Yang H. F. Li Y. J. Chen Y. M. (2019). Effects of the intervention of wnt/beta-catenin signaling pathway on skin tissue fibrosis and epithelial-mesenchymal transition in mouse models of scleroderma. Zhongguo Zuzhi Gongcheng Yanjiu 23 (23), 3680–3685.

[B40] Liu S. Yang B. Yang P. Liu Z. (2019). Herbal Formula-3 ameliorates OVA-induced food allergy in mice may via modulating the gut microbiota. Am. J. Transl. Res. 11 (9), 5812–5823. 31632550 PMC6789213

[B41] Luo X. Y. Yang Z. H. (2018). Research progress on the correlation between gut microbiota and cardiovascular diseases. Chin. Pharmacol. Bull. 34 (08), 1037–1041.

[B42] Mandujano A. Méndez-Ramírez I. Silveira-Torre L. H. (2021). Systemic sclerosis: elevated levels of leukotrienes in saliva and plasma are associated with vascular manifestations and nailfold capillaroscopic abnormalities. Int. J. Environ. Res. Public Health 18 (20), 10841. 10.3390/ijerph182010841 34682587 PMC8536043

[B43] Mao C. F. Sun H. Zou Q. H. Shi Y. Yang L. Wan P. (2020). Microecology of tongue coating flora in patients with rheumatoid arthritis. J. Army Med. Univ. 42 (07), 708–713. 10.16016/j.1000-5404.201912166

[B44] Morales-González V. Galeano-Sánchez D. Covaleda-Vargas J. E. Rodriguez Y. Monsalve D. M. Pardo-Rodriguez D. (2023). Metabolic fingerprinting of systemic sclerosis: a systematic review. Front. Mol. Biosci. 10, 1215039. 10.3389/fmolb.2023.1215039 37614441 PMC10442829

[B45] Morin F. Kavian N. Batteux F. (2015). Animal models of systemic sclerosis. Curr. Pharm. Des. 21 (18), 2365–2379. 10.2174/1381612821666150316120338 25777753

[B46] Murgia F. Svegliati S. Poddighe S. Lussu M. Manzin A. Spadoni T. (2018). Metabolomic profile of systemic sclerosis patients. Sci. Rep. 8 (1), 7626. 10.1038/s41598-018-25992-7 29769578 PMC5955890

[B47] Nakashima S. Kakugawa T. Yura H. Tomonaga M. Harada T. Hara A. (2015). Identification of *Helicobacter pylori* VacA in human lung and its effects on lung cells. Biochem. Biophysical Res. Commun. 460 (3), 721–726. 10.1016/j.bbrc.2015.03.096 25817795 PMC6118350

[B48] Narsing Rao M. P. Luo Z. H. Dong Z. Y. Li Q. Liu B. B. Guo S. X. (2022). Metagenomic analysis further extends the role of chloroflexi in fundamental biogeochemical cycles. Environ. Res. 209, 112888. 10.1016/j.envres.2022.112888 35143804

[B49] Olman M. A. White K. E. Ware L. B. Simmons W. L. Benveniste E. N. Zhu S. (2004). Pulmonary edema fluid from patients with early lung injury stimulates fibroblast proliferation through IL-1 beta-induced IL-6 expression. J. Immunol. Baltim. Md 172 (4), 2668–2677. 10.4049/jimmunol.172.4.2668 14764742

[B50] Orlandi M. Lepri G. Damiani A. Barsotti S. Di Battista M. Codullo V. (2020). One year in review 2020: systemic sclerosis. Clin. Exp. Rheumatology 38 (3), 3–17. 32662404

[B51] Orlandi M. Lepri G. Di Battista M. De Mattia G. Da Rio M. Codullo V. (2022). Systemic sclerosis: one year in review 2022. Clin. Exp. Theumatology 40 (10), 1911–1920. 10.55563/clinexprheumatol/3401fl 36135958

[B52] Park M. J. Park Y. Choi J. W. Baek J. A. Jeong H. Y. Na H. S. (2022). Establishment of a humanized animal model of systemic sclerosis in which T helper-17 cells from patients with systemic sclerosis infiltrate and cause fibrosis in the lungs and skin. Exp. and Mol. Med. 54 (9), 1577–1585. 10.1038/s12276-022-00860-7 36175484 PMC9534900

[B53] Punjasamanvong S. Muangchan C. (2021). Persistent eosinophilia and associated organ involvement in Thai patients with systemic sclerosis: data from the siriraj scleroderma cohort. Archives Rheumatology 36 (4), 527–537. 10.46497/ArchRheumatol.2021.8855 35382361 PMC8957775

[B54] Romanowska-Próchnicka K. Dziewit M. Lesiak A. Reich A. Olesińska M. (2024). Scleroderma and scleroderma-like syndromes. Front. Immunol. 15, 1351675. 10.3389/fimmu.2024.1351675 38887288 PMC11180720

[B55] Ross R. L. Mavria G. Del Galdo F. Elies J. (2022). Downregulation of vascular Hemeoxygenase-1 leads to vasculopathy in systemic sclerosis. Front. Physiology 13, 900631. 10.3389/fphys.2022.900631 35600300 PMC9117635

[B56] Sachar M. Anderson K. E. Ma X. (2016). Protoporphyrin IX: the good, the bad, and the ugly. J. Pharmacol. Exp. Ther. 356 (2), 267–275. 10.1124/jpet.115.228130 26588930 PMC4727154

[B57] Sarhan S. A. El-Meligui Y. M. (2023). Significance of platelets to lymphocytes and platelets to haemoglobin ratios in patients with systemic sclerosis. Reumatol. Clin. 19 (1), 12–17. 10.1016/j.reumae.2021.11.005 36603962

[B58] Scher J. U. Sczesnak A. Longman R. S. Segata N. Ubeda C. Bielski C. (2013). Expansion of intestinal Prevotella copri correlates with enhanced susceptibility to arthritis. eLife 2, e01202. 10.7554/eLife.01202 24192039 PMC3816614

[B59] Schrimpe-Rutledge A. C. Codreanu S. G. Sherrod S. D. McLean J. A. (2016). Untargeted metabolomics strategies-challenges and emerging directions. J. Am. Soc. Mass Spectrom. 27 (12), 1897–1905. 10.1007/s13361-016-1469-y 27624161 PMC5110944

[B60] Stone T. W. Darlington L. G. Badawy A. A. Williams R. O. (2024). The complex world of kynurenic acid: reflections on biological issues and therapeutic strategy. Int. J. Mol. Sci. 25 (16), 9040. 10.3390/ijms25169040 39201726 PMC11354734

[B61] Teaw S. Hinchcliff M. Cheng M. (2021). A review and roadmap of the skin, lung and gut microbiota in systemic sclerosis. Rheumatol. Oxf. Engl. 60 (12), 5498–5508. 10.1093/rheumatology/keab262 33734316 PMC8643452

[B62] Tett A. Pasolli E. Masetti G. Ercolini D. Segata N. (2021). Prevotella diversity, niches and interactions with the human host. Nat. Rev. Microbiol. 19 (9), 585–599. 10.1038/s41579-021-00559-y 34050328 PMC11290707

[B63] Tian J. Kang S. Zhang D. Huang Y. Zhao M. Gui X. (2023). Global, regional, and national incidence and prevalence of systemic sclerosis. Clin. Immunol. Orl. Fla. 248, 109267. 10.1016/j.clim.2023.109267 36804224

[B64] Turska-Kozłowska M. Pedraz-Petrozzi B. Paluszkiewicz P. Parada-Turska J. (2024). Different kynurenine pathway dysregulation in systemic sclerosis in men and women. Int. J. Mol. Sci. 25 (7), 3842. 10.3390/ijms25073842 38612652 PMC11011858

[B65] Van Averbeke V. Berkell M. Mysara M. Rodriguez-Ruiz J. P. Xavier B. B. De Winter F. H. R. (2022). Host immunity influences the composition of murine gut microbiota. Front. Immunol. 13, 828016. 10.3389/fimmu.2022.828016 35371073 PMC8965567

[B66] Wang B. Wu L. Chen J. Dong L. Chen C. Wen Z. (2021). Metabolism pathways of arachidonic acids: mechanisms and potential therapeutic targets. Signal Transduct. Target. Ther. 6 (1), 94. 10.1038/s41392-020-00443-w 33637672 PMC7910446

[B67] Wang Y. Liu Z. Shen P. Zhao C. Liu B. Shu C. (2022). Kynurenic acid ameliorates lipopolysaccharide-induced endometritis by regulating the GRP35/NF-κB signaling pathway. Toxicol. Appl. Pharmacol. 438, 115907. 10.1016/j.taap.2022.115907 35123988

[B68] Wirthgen E. Hoeflich A. Rebl A. Günther J. (2018). Kynurenic acid: the janus-faced role of an immunomodulatory tryptophan metabolite and its link to pathological conditions. Front. Immunol. 8, 1957. 10.3389/fimmu.2017.01957 29379504 PMC5770815

[B69] Wu X. Cai X. Ai J. Zhang C. Liu N. Gao W. (2021). Extraction, structures, bioactivities and structure-function analysis of the polysaccharides from safflower (Carthamus tinctorius L.). Front. Pharmacol. 12, 767947. 10.3389/fphar.2021.767947 34744747 PMC8563581

[B70] Xing C. Wang M. Ajibade A. A. Tan P. Fu C. Chen L. (2021). Microbiota regulate innate immune signaling and protective immunity against cancer. Cell Host and Microbe 29 (6), 959–974.e7. 10.1016/j.chom.2021.03.016 33894128 PMC8192480

[B71] Yamamoto T. Takagawa S. Katayama I. Yamazaki K. Hamazaki Y. Shinkai H. (1999). Animal model of sclerotic skin. I: local injections of bleomycin induce sclerotic skin mimicking scleroderma. J. Investigative Dermatology 112 (4), 456–462. 10.1046/j.1523-1747.1999.00528.x 10201529

[B72] Yang H. H. Lyu J. Y. (2013). Literature analysis on Chinese herbal medicine for the treatment of systemic sclerosis. Rheumatism Arthritis 2 (03), 36–37+48.

[B73] Zhang T. Ji X. Lu G. Zhang F. (2021). The potential of Akkermansia muciniphila in inflammatory bowel disease. Appl. Microbiol. Biotechnol. 105 (14-15), 5785–5794. 10.1007/s00253-021-11453-1 34312713

