# Supplementary material for: Exploring the anti-fibrotic effects of safflower in systemic sclerosis based on metabolomics and gut microbiota analyses
Source: Front Pharmacol. 2026 Feb 18;17:1719219. doi: 10.3389/fphar.2026.1719219 (PMC12957090; doi:10.3389/fphar.2026.1719219)
Supplement: Supplementary file 3 [file DataSheet1.pdf]

1. Method

1.1 Chromatographic conditions

The separation of sample was achieved on an ACQUITY UPLC®HSS T3 column (100 mm × 2.1 mm, 1.7 μm); The mobile phase consists of 0.1% formic acid in an aqueous solution (A) and acetonitrile (B) and the gradient program as the following: 0~0.5min，2% B；0.5~1min，2%~5% B；1~4.5min，5%~40% B；4.5~5.5min，40%~80% B；5.5~7min，80%~98% B；7~8min，98%~2% B；8~10min，2% B；The flow rate was 0.40 mL/min. The column temperature was 40 ℃. The injection volume was 3μL.

1.2 Mass spectrometry conditions

The optimized condition was as follows: source temperature at 80℃(ESI^+) and 100℃ (ESI^-), desolvation gas flow of 400 L/h at 450℃(ESI^+) and 600 L/h at 250℃ (ESI^-), cone gas flow at 30 L/h(ESI^+) and 50 L/h (ESI^-), capillary voltage of 3.0 kV (ESI^+) and 2.5 kV (ESI^-), sampling cone voltage of 40 V. MS data were acquired in full scan mode from m/z 50 to m/z 1500 at acquisition rate of 0.3 s/scan, the calibrant solution, leucine-enkephalin (400 ng/mL), was continuously introduced to MS system at a flow-rate of 20μL/min via the LockSpray™ interface to ensure the accuracy and reproducibility of TOFMS.

1.3 Identification method

Based on the combination of fragment ions with literature data and public database (Human Metabolome database and Chemicalbook), a total of 20 compounds were identified from the aqueous extract of Safflower.

2. Result

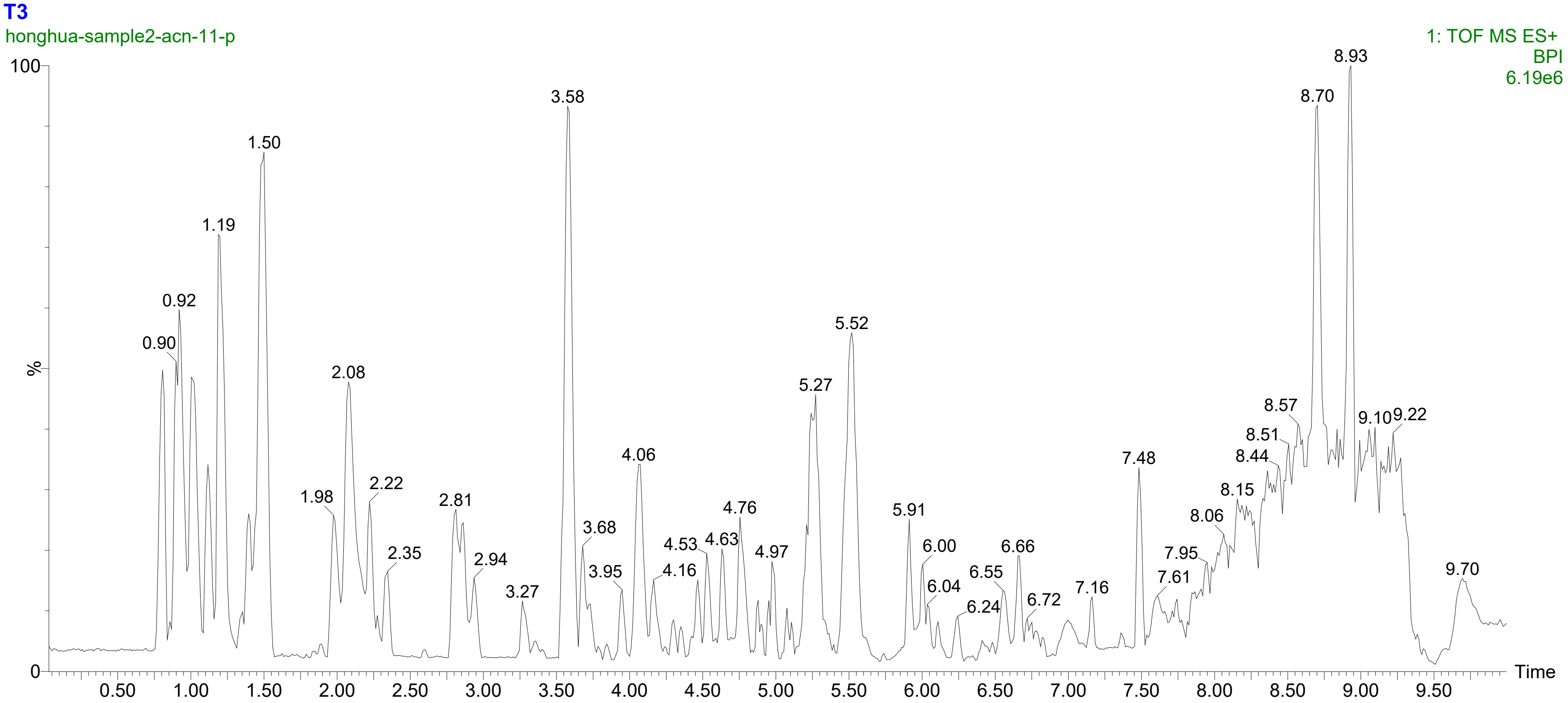

Figure 1 .

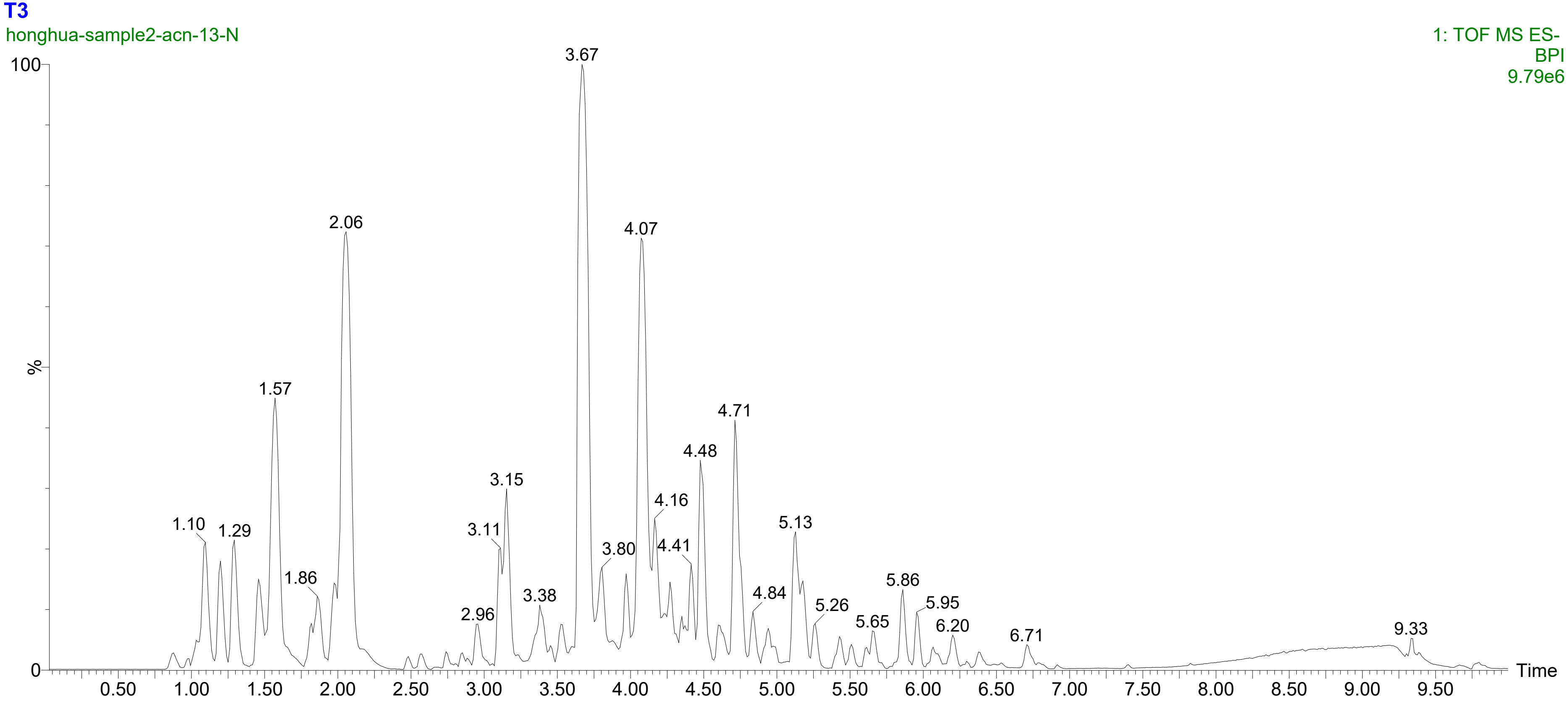
T3
honghua-sample2-acn-13-N
1: TOF MS ES-
BPI
9.79e6
100
%
0
3.67
2.06
4.07
1.57
4.71
4.48
3.15
4.16
5.13
1.10
1.29
3.11
3.80
4.41
1.86
3.38
4.84
5.86
5.26
5.95
2.96
5.65
6.20
6.71
9.33
0.50
1.00
1.50
2.00
2.50
3.00
3.50
4.00
4.50
5.00
5.50
6.00
6.50
7.00
7.50
8.00
8.50
9.00
9.50
Time

Figure 2 .

Table 1. Results of qualitative identification

Peak no.	RT (min)	Quasi-molecular ion (measured)	Formula	Ion type (measured)	Error (ppm)	MS^n data (measured)	Identification
1	2.85	625.1383	$C_{27}H_{30}O_{17}$	M-H	3.5	625.1406;607.1299;535.1075	Quercetin 4',7-diglucoside
2	2.96	463.0873	$C_{21}H_{20}O_{12}$	M-H	0.9	463.0873;445.0762;407.0925	Quercetin 4'-glucoside
3	3.15	491.1179	$C_{23}H_{24}O_{12}$	M-H	2.2	491.1183;473.1071;461.1052	3',8-Dimethoxyapigenin 7-glucoside
4	3.15	435.0907	$C_{20}H_{20}O_{11}$	M-H	4.6	435.0915;417.0838;407.0963	Homomangiferin
5	3.38	625.1390	$C_{27}H_{30}O_{17}$	M-H	2.4	625.1401;607.1285;463.0867	Quercetin 3,4'-diglucoside
6	3.52	611.1600	$C_{27}H_{32}O_{16}$	M-H	2.0	611.1612;593.1503;449.1075	Hydroxysafflor yellow A
7	4.078	593.1513	$C_{27}H_{30}O_{15}$	M-H	1.2	593.1505;579.1392;505.0990;489.1013	Biorobin
8	4.41	463.0893	$C_{21}H_{20}O_{12}$	M-H	3.5	463.0870;445.0772;301.0340	Isoquercitrin
9	4.48	609.1451	$C_{27}H_{30}O_{16}$	M-H	0.8	612.1547;611.1518;610.1494;609.1459	Rutin
10	4.84	449.1073	$C_{21}H_{22}O_{11}$	M-H	2.4	449.1082;287.0551	Miscanthoside
11	5.13	613.1613	$C_{30}H_{30}O_{14}$	M-H	9.0	613.1557;595.1453;569.1666;551.1549	Safflomin C
12	5.13	505.1335	$C_{24}H_{26}O_{12}$	M-H	2.2	505.1311;487.1291;475.1219	Caryatin glucoside
13	5.65	557.1299	$C_{27}H_{26}O_{13}$	M-H	0.7	557.1301;593.1194	Piceatannol 4'-galloylglucoside
14	3.27	303.0503	$C_{15}H_{10}O_{7}$	M+H	0.7	303.0495;287.0191;277.0356	Quercetin
15	3.58	433.1123	$C_{21}H_{20}O_{10}$	M+H	2.8	433.1112;415.1006;397.0902	Cosmosiin
16	4.06	595.1703	$C_{27}H_{30}O_{15}$	M+H	6.7	593.1669;577.1552;559.1402	Safflor Yellow A
17	4.16	473.1063	$C_{23}H_{20}O_{11}$	M+H	4.4	473.1036;455.0980;289.0703	(-)-Epigallocatechin 3-(4-methyl-gallate)
18	4.53	1045.2904	$C_{48}H_{52}O_{26}$	M+H	7.6	1045.2878;971.2484	Anhydrosafflor Yellow B
19	4.76	287.0569	$C_{15}H_{10}O_{6}$	M+H	4.5	287.0546;269.0470;257.0454	Kaempferol
20	5.24	289.0730	$C_{15}H_{12}O_{6}$	M+H	6.2	289.0703;273.0399;259.0670	Eriodictyol
